# Collision of Hypopharyngeal Small‐Cell Neuroendocrine Carcinoma and Laryngeal Squamous Cell Carcinoma at the Aryepiglottic Fold: Case Report and Review of Literature

**DOI:** 10.1155/crip/6630405

**Published:** 2026-04-10

**Authors:** Neda Mladenovic, Sasa Jakovljevic, Nada Tomanovic, Katarina Jovanovic, Nemanja Radivojevic, Djurdjina Kablar, Ana Marija Tomic

**Affiliations:** ^1^ Clinic for Otorhinolaryngology and Maxillofacial Surgery, University Clinical Center of Serbia, Belgrade, Serbia, kcs.ac.rs; ^2^ Faculty of Medicine, University of Belgrade, Belgrade, Serbia, bg.ac.rs; ^3^ Institute of Pathology, Belgrade, Serbia, klinikum-fuerth.de; ^4^ Department for Pathology, Pathohistology and Medical Cytology, University Clinical Centre of Serbia, Belgrade, Serbia, kcs.ac.rs

**Keywords:** collision tumor, immunohistochemistry, larynx, neuroendocrine carcinoma, squamous cell carcinoma

## Abstract

**Background:**

Collision tumors are neoplasms in which two histologically different and topographically independent tumors are joined within the same mass. The aim of this paper is to present a rare case of collision carcinoma of the larynx (squamous cell and small‐cell neuroendocrine carcinoma).

**Case Report—Diagnosis/Treatment:**

A 72‐year‐old patient was reported to our clinic because of hoarseness, dyspnea, and dysphagia. Neck CT showed a right‐sided laryngeal tumor. The patient underwent total laryngectomy, partial pharyngectomy, and right‐sided thyroidectomy with bilateral selective neck dissection. A clear collision of two morphologically and immunohistochemically different components of the tumor was seen on the sections sampled from the aryepiglottic fold.

**Conclusions:**

This case highlights the critical importance of obtaining multiple samples during tumor biopsy and emphasizes the role of detailed immunohistochemical staining. Considering the differences in histology, it is difficult to propose guidelines for treatment of these cancers.

## 1. Introduction

Laryngeal carcinoma is the third most common malignancy occurring in the head and neck region worldwide, with a male to female ratio of approximately 7:1 [1]. The majority of these tumors are of squamous cell origin (about 95% of all cases) with the glottis as the most common site of involvement [1, 2]. Neuroendocrine carcinomas of the larynx are extremely rare, comprising about 0.5% of all laryngeal tumors and are most often found in the supraglottis [2, 3].

Collision tumors are defined differently in the literature and final compromise has not yet been reached regarding their definition. The accepted definition is that they are neoplasms in which two histologically different and topographically independent tumors are joint within the same mass [3]. They can be found in all parts of the body, including the head and neck region, in which they are very rare. The most common location in this region is the thyroid gland. Very few cases of laryngeal collision tumors composed of squamous cell carcinoma and small‐cell neuroendocrine carcinoma have been reported in the literature, highlighting the rarity of this entity [3, 4, 5].

The aim of this paper was to present an extremely rare case of collision carcinoma of the larynx (squamous cell and small‐cell neuroendocrine carcinoma) in a 72‐year‐old patient.

## 2. Case Report

A 72‐year‐old patient was referred to the otolaryngology department because of hoarseness, dyspnea, and dysphagia. The symptoms were present for 3 months. Indirect laryngoscopy revealed a tumorous mass of the right hemilarynx spreading to the right pyriform sinus and the right lateral wall of the hypopharynx. On the right side of the neck in the Regions II and IV, painless enlarged lymph nodes about 2.5 cm in size were noted. The patient′s medical history was notable for diabetes and bilateral carotid artery surgery following a stroke in 2011. Other findings in the head and neck region were normal. A computerized tomography (CT) of the neck was performed, which described a tumor affecting the supraglottic, glottic, and partly the subglottic part of the larynx on the right side. The tumor was found in the right pyriform sinus and the right aryepiglottic fold as well. The size of the lesion on the CT scan was 31 × 39 × 38 mm. (Figure 1a, b) Enlarged lymph nodes on the right side of the neck were described in the IV region with a size of 20 × 18 × 20 mm and in the IIa region, sized 20 × 17 × 22 mm.

Figure 1(a–c) (a) CT neck, axial view, arrow indicates tumor mass; (b) CT neck, coronal view, arrow indicates tumor mass; (c) intraoperative sample of the larynx, blue arrow indicates squamous cell carcinoma on the laryngeal side of the epiglottis; black arrow indicates neuroendocrine carcinoma.

**Figure:**
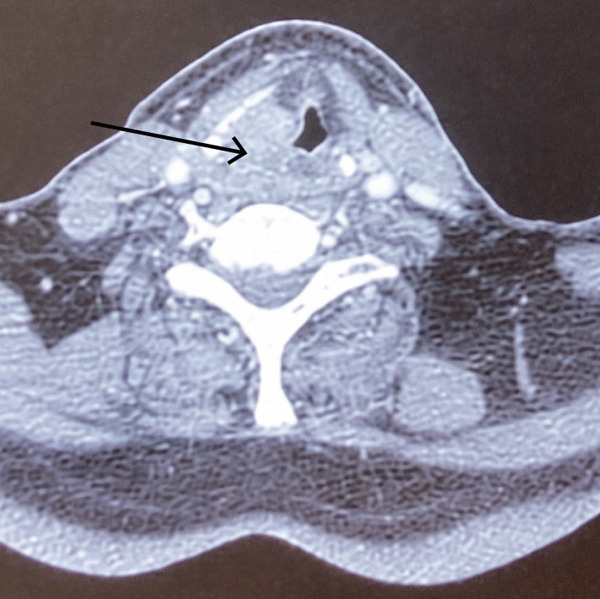
(a)

**Figure:**
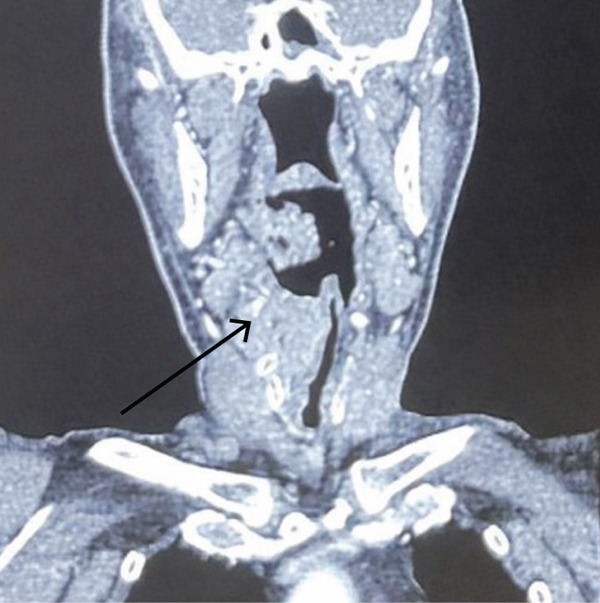
(b)

**Figure:**
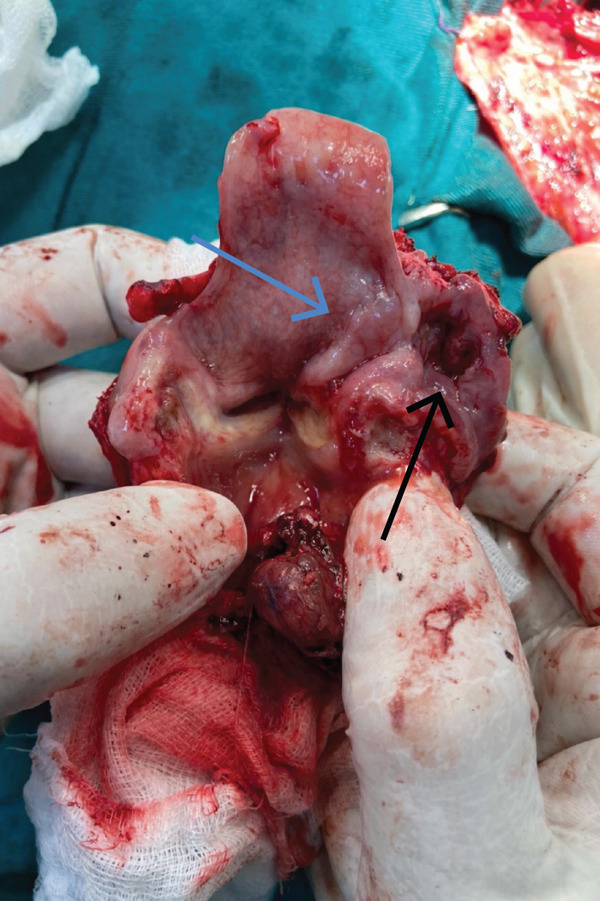
(c)

Chest radiography and ultrasound of the abdomen were unremarkable. The patient underwent surgical tracheotomy due to dyspnea. Consecutively, laryngomicroscopy was performed, during which an ulcerovegetative tumor was described. The tumor was found to dominantly affect the laryngeal side of the epiglottis, the right pyriform sinus, and the right aryepiglottic and ventricular fold. Two samples were taken for pathohistological analysis (the first sample from the laryngeal side of the epiglottis, and the second from the right pyriform sinus). After pathohistological and immunohistochemical (IHC) analysis, it was established that the tumor lesion on the laryngeal side of the epiglottis corresponds to squamous cell carcinoma. The finding in the sample taken from the pyriform sinus on the right was found to correspond to mucosal infiltration of a small‐cell neuroendocrine carcinoma. The pathologist′s conclusion was that these two tumors differ morphologically and IHC and that a detailed clinical–pathological correlation was required. The patient was referred to the Council for Malignant Diseases, which made the decision to carry out operative treatment. The patient underwent total laryngectomy with partial pharyngectomy and right‐sided thyroidectomy with bilateral selective neck dissection. (Figure 1c) Pathohistological and IHC analysis of the dissections from the right side of the neck showed that 11 of the 17 lymph nodes contained metastases of poorly differentiated neuroendocrine carcinoma. There were no metastases in the lymph nodes on the left side. IHC profiles of epiglottic tumor and pyriform sinus tumor are shown in Table 1. (Figure 2a, 2b, and 2c) The neuroendocrine component showed poorly differentiated morphology with a diffuse expression of neuroendocrine markers, 17 mitoses per 2 mm [2] and Ki‐67 proliferative index exceeding 90%, fulfilling the WHO 2022 criteria for small‐cell neuroendocrine carcinoma [6]. Two components were topographically independent, showed a sharp interface without transitional areas, and retained completely distinct morphological and IHC profiles, so mixed neuroendocrine–non‐neuroendocrine neoplasm was excluded.

**Table 1:** Immunohistochemical profile of the two tumor components.

IHC stain	Epiglottic tumor	Pyriform sinus tumor
CK	+	+
P40	+	−
CK5/6	+	−/+
CK7	−	−/+
Synaptophysin	−	+
Chromogranin A	−	+
INSM1	−	+
Ki‐67^a^	40%	90%

Abbreviation: IHC, immunohistochemical.

^a^Areas of highest nuclear staining (“hot spots”) were identified at low magnification; Ki‐67 proliferation index was then calculated by manual counting of at least 500 tumor cell nuclei in the selected hot spot areas at high power magnification, and expressed as the percentage of tumor cell nuclei showing unequivocal nuclear positivity.

Figure 2(a–c) (a) Immunohistochemical staining for p40 and synaptophysin antibody indicative of collision point between the two distinct tumor components: squamous cell carcinoma (on the left) shows intensive and diffuse nuclear p40 positivity, whereas synaptophysin stain remains negative; neuroendocrine carcinoma (on the right) shows diffuse and moderate synaptophysin stain positivity, whereas p40 stain remains negative. Streptavidin–biotin, original magnification ×400. (b) Immunohistochemical staining for cytokeratin with emphasis on the collision point of two distinct histopathological components: squamous cell carcinoma and neuroendocrine carcinoma; there are two distinct cytokeratin staining patterns between squamous cell carcinoma (intensive membranous and cytoplasmic positivity) and neuroendocrine carcinoma (“dot‐like” cytoplasmic pattern of staining). Streptavidin–biotin, original magnification × 400. (c) Immunohistochemical staining for Ki‐67 antibody indicating different proliferative indexes in two distinct collision tumor components: neuroendocrine carcinoma (on the right) has more than 90% of Ki‐67 positive nuclei, whereas squamous cell carcinoma has a much lower proliferative index of up to 40% positive nuclei. Streptavidin–biotin, original magnification ×400.

**Figure:**
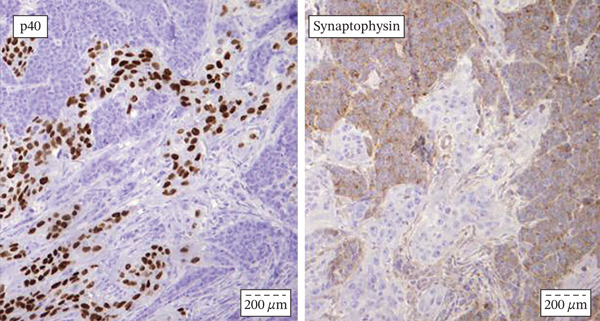
(a)

**Figure:**
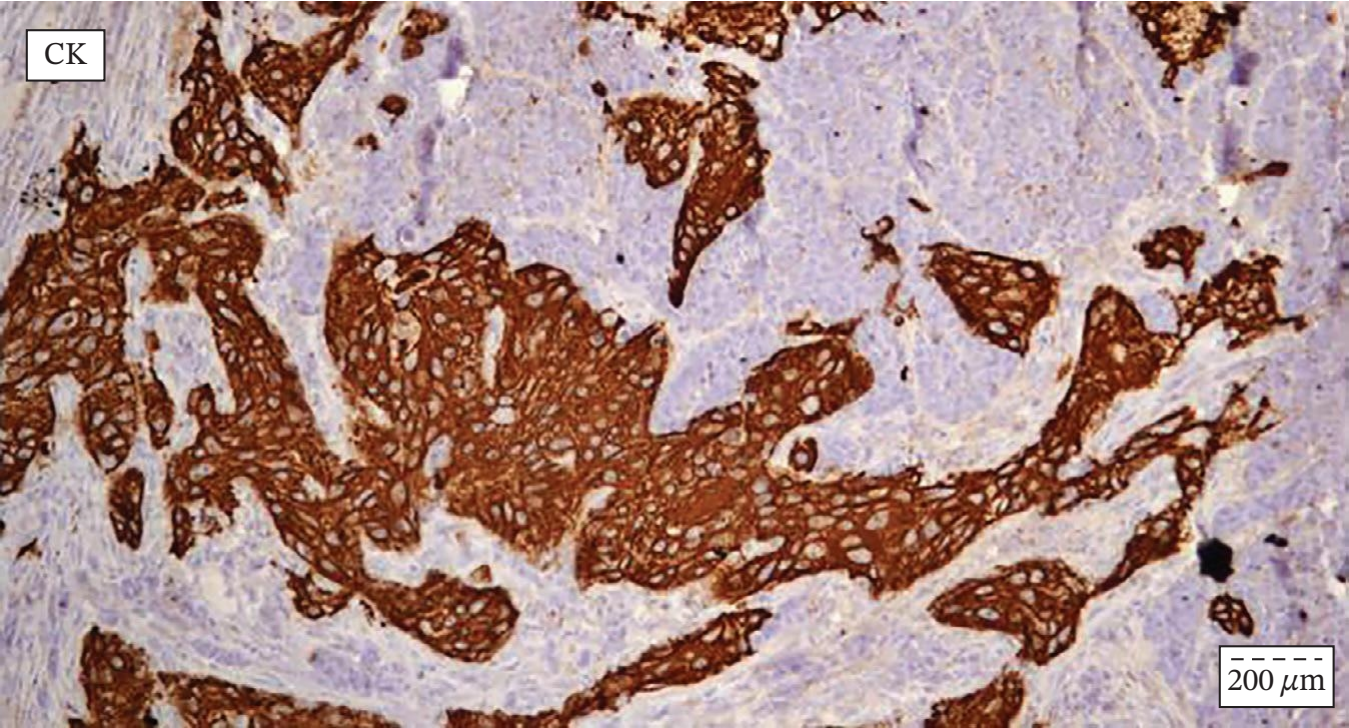
(b)

**Figure:**
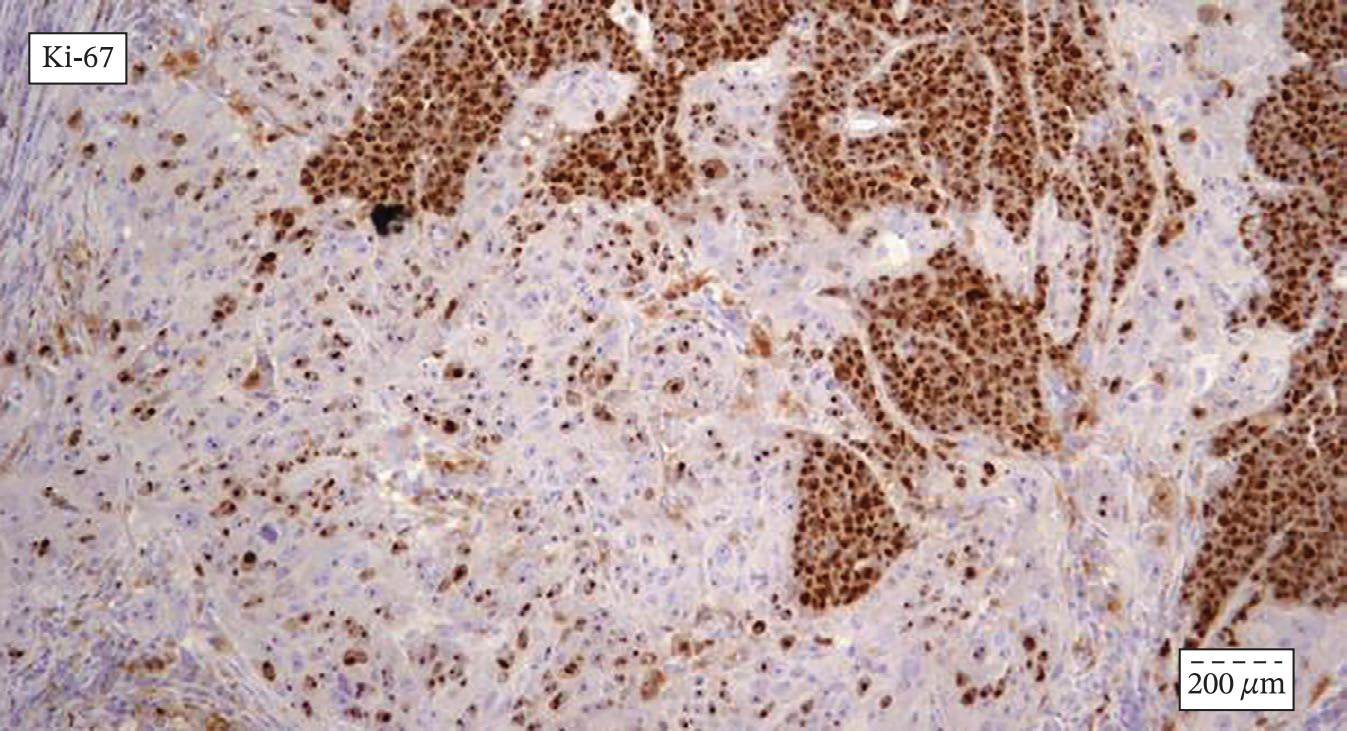
(c)

On the sections sampled from the region of the aryepiglottic fold, a clear collision of two morphologically and IHC different components of the tumor was seen. (Figure 3a, b) It was concluded that it was indeed a collision tumor of the larynx: squamous cell carcinoma and small‐cell neuroendocrine carcinoma. The patient was once again presented to the Council for Malignant Diseases, which decided to continue the treatment with postoperative radiotherapy with chemotherapy potentiation. A total dose of 66 Gy was administered in 33 fractions with three cycles of cisplatin. At the first follow‐up 6 weeks after the postoperative radiotherapy and chemotherapy, no signs of disease recurrence were noted.

Figure 3(a, b) (a) Collision tumor with two distinct histopathological components: squamous cell carcinoma (left) and small cell neuroendocrine carcinoma (right). Hematoxylin and eosin, original magnification × 200. (b) Detail of collision tumor point with two distinct histopathological components: squamous cell carcinoma (left) and small cell neuroendocrine carcinoma (right). Hematoxylin and eosin, original magnification × 400.

**Figure:**
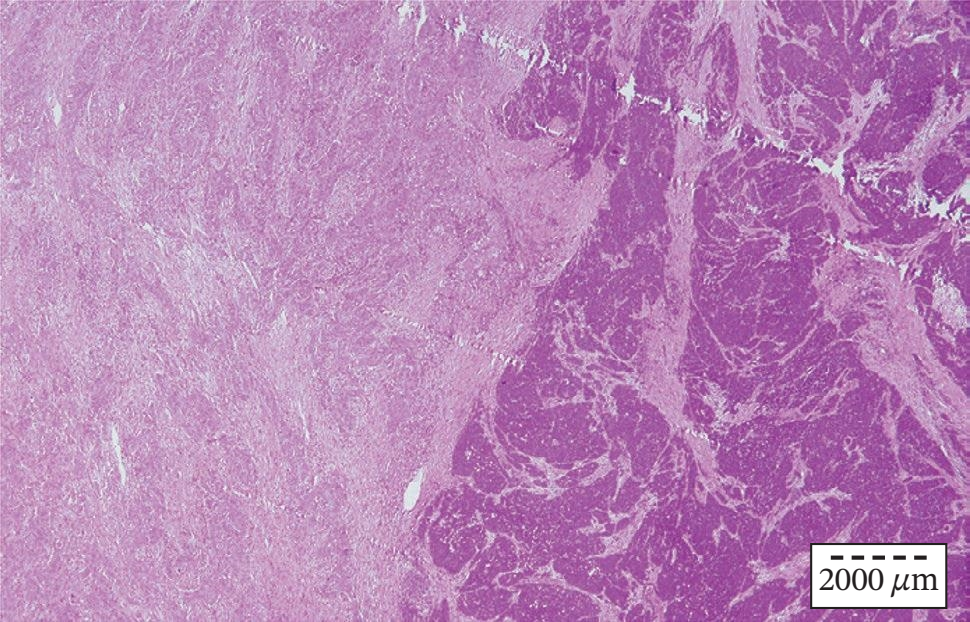
(a)

**Figure:**
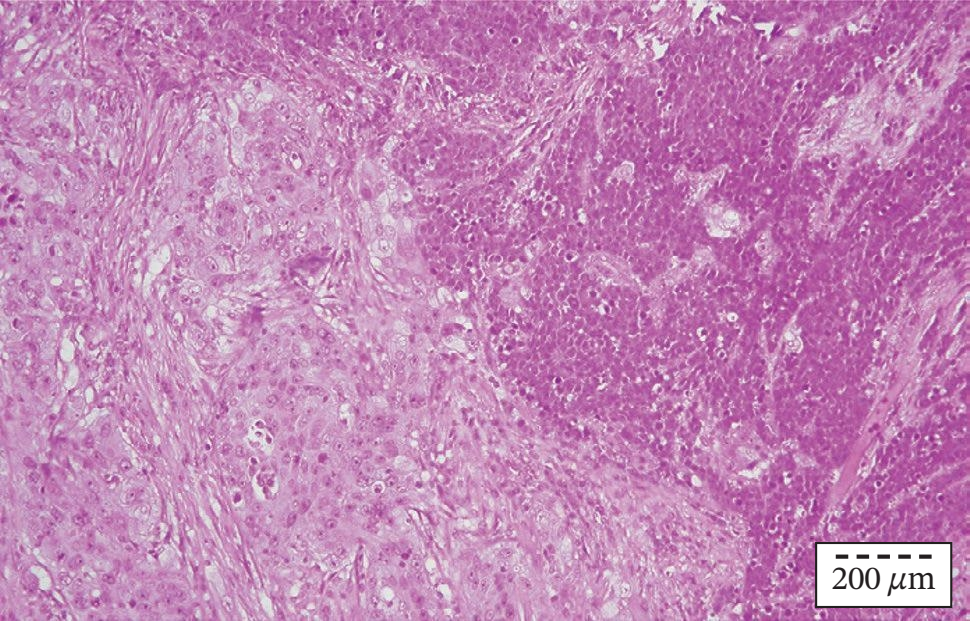
(b)

## 3. Discussion

This literature review was conducted using the PubMed and Scopus databases. The search included articles published up to January 2026 (key words: “laryngeal collision tumor,” “squamous cell carcinoma and neuroendocrine carcinoma,” “laryngeal small cell neuroendocrine carcinoma,” and “combined laryngeal malignancies”). Only English‐language case reports and reviews were included.

The definition of collision tumors is still debated, but according to the definition given by Spagnolo and Heenan, they are usually defined as the coexistence of two histologically and morphologically different tumors within the same anatomical area, without histological admixtures at their interface. They can be composed of two primary tumors from the same organ, or a primary tumor and a metastasis, and must be distinguished from a composite tumor, where two different histological types are completely mixed [4, 5, 7, 8]. Namely, composite tumors also include two morphologically and IHC different neoplasms coexisting near the same organ. However, they present with an actual cellular mixture and a common mutation that results in divergent histology from a common source [8, 9].

The first case of combined neuroendocrine and squamous cell carcinoma of the larynx was described and reported in 1985 [1]. Upon reviewing previously reported cases of collision tumors of the larynx, we found that squamous cell carcinoma was a common component in all cases, whereas the neuroendocrine component was small‐cell carcinoma in all but one case, which was an atypical carcinoid [10]. None of the cases reported in the literature presented with a paraneoplastic syndrome.

Although early literature on laryngeal collision tumors cited only sporadic cases, more recent analyses have expanded our understanding of this rare entity. One report summarized 12 cases of squamous cell carcinoma and neuroendocrine carcinoma collision tumors in the head and neck region, highlighting the challenges in diagnosis and treatment due to dual histology and limited experience [4]. A larger review of 27 cases of squamous cell carcinoma and neuroendocrine carcinoma collisions in the head and neck region found that most tumors originated in the larynx and emphasized variable treatment strategies and outcomes [11]. Furthermore, a recent review of neuroendocrine carcinomas of the larynx reinforces the rarity and diagnostic complexity of these tumors, even outside a collision context [12].

The exact pathophysiological mechanism of collision tumor development is still unknown; however, several theories have been proposed in the literature. The widely accepted theory is that collision tumors express neoplastic heterogeneity, which indicates that they develop as a result of two different clones of neoplastic cells [13]. Within this theory, several mechanisms have been proposed, the first of which is pure coincidence. Another possible mechanism is the theory of field carcinogenesis, which implies that parts of the tissue exposed to repeated damage by common carcinogens have an increased chance of developing two separate neoplasms in one location. A third possible mechanism is the interaction theory, which suggests that one neoplasm produces epithelial or stromal changes that further induce the formation of another independent neoplasm through paracrine effects [4, 7, 9, 13].

These tumors can be difficult to diagnose and a thorough pathological analysis, including IHC staining of the specimen is required in order to set an accurate diagnosis [1, 7]. On the other hand, the preoperative diagnostic accuracy of these tumors is very poor since it is very difficult to diagnose both histological types on a single specimen [5]. Therefore, a precise and detailed examination of samples taken during laryngectomy is very important [14]. We suggest that collision carcinoma should be considered only when there are two malignant tumors that look similar to the naked eye and originate from the same organ, but are of different pathological types and neither of the neoplasms migrated to another [4]. A recent study analyzing the staining characteristics of neuroendocrine carcinomas showed that the most reliable typical staining is synaptophysin and chromogranin A [1]. In our case, the sample staining was positive for both. The article also noted that positive IHC staining for synaptophysin and chromogranin A occurs in neuroendocrine tumors regardless of their location. However, another study showed that the two most common IHC stains for neuroendocrine tumors—synaptophysin and chromogranin A—were present in head and neck tumor samples in only 41% and 18% of cases, respectively [1]. The difference in the study results emphasizes the complexity surrounding the diagnostic process of collision tumors.

It is generally accepted that the treatment of collision carcinoma should be based on the treatment of more invasive or malignant histology [4, 15]. According to the National Comprehensive Cancer Network guidelines (ver. 1.2015) [16], patients with poorly differentiated neuroendocrine carcinomas, such as our case, can be divided into in three treatment groups: [1] recommended treatment for resectable tumors is resection + chemotherapy ± radiotherapy or definitive chemoradiotherapy is considered, [2] recommended treatment for locoregional unresectable tumors is radiotherapy + chemotherapy, and [3] recommended treatment for tumors with distant metastases is only chemotherapy [8]. Considering these recommendations, our patient underwent operative treatment with postoperative radiotherapy.

The clinical course of all reported cases in the literature was fatal with an early occurrence of distant metastases [2]. Disease prognosis in patients with laryngeal collision tumors is considered to be determined by the prognosis of the more aggressive tumor. In our case, the neuroendocrine carcinoma showed a more advancing course. The commanding prognostic influence of neuroendocrine carcinomas in collision tumors is seen probably due to their faster growth and higher metastatic potential compared with squamous cell carcinoma [14]. Almost half of the patients present with positive cervical lymph nodes, our patient included. About 60%–90% of the patients develop distant metastases [3, 8, 14]. Despite advanced cancer treatment, 2‐ and 5‐year survival rates are only 16% and 5%, respectively [3, 10]. Because small‐cell neuroendocrine carcinoma is characterized by a high propensity for distant metastases early in the disease course, comprehensive staging including whole body PET/CT is generally recommended in order to exclude systemic disease and guide further treatment. In our case, PET/CT was not performed as it is not available as a routine preoperative diagnostic modality at our institution. Staging consisted of neck CT, chest radiography and abdominal ultrasound, which did not reveal distant metastases. The absence of PET/CT imaging represents a limitation of this report.

Another limitation of this case report is the short duration of postoperative follow‐up. Although the patient showed no evidence of disease recurrence 6 weeks after completion of adjuvant chemoradiotherapy, longer follow‐up would be necessary to better assess oncologic outcomes. Unfortunately, extended follow‐up data could not be obtained, as the patient did not continue oncologic surveillance at our institution. Given the high metastatic potential of small cell neuroendocrine carcinoma, regular postoperative surveillance is generally recommended. This typically includes clinical examination and flexible endoscopy every 1–3 months during the first year, combined with periodic cross‐sectional imaging of the neck and chest to allow early detection of locoregional recurrence or distant metastases.

## 4. Conclusion

There are still numerous controversies surrounding the definition, diagnosis, and treatment of laryngeal collision tumors, primarily due to their low incidence. The patient presented in this study highlights the critical importance of obtaining multiple samples during tumor biopsy and emphasizes the role of detailed IHC staining of head and neck tumor specimens. Considering the differences in histology, it is difficult to propose guidelines for the treatment of these cancers, and multimodal treatment is suggested with consideration of both histological and functional outcomes.

## Funding

No funding was received for this manuscript.

## Ethics Statement

Ours is a medical record‐based case report for which institute ethical committee acceptance is not applicable, and hence, was not applied for. In our institute, case reports are not presented for ethical committee clearance. Informed consent was obtained from the participant included in the case report.

## Conflicts of Interest

The authors declare no conflicts of interest.

## Data Availability

The data used to write this case report are available upon request from the corresponding author. The data are not publicly available due to privacy or ethical restrictions.
